# Supplementary material for: Across atoms to crossing continents: Application of similarity measures to biological location data
Source: PLoS One. 2023 May 15;18(5):e0284736. doi: 10.1371/journal.pone.0284736 (PMC10184918; doi:10.1371/journal.pone.0284736)
Supplement: S2 File — Additional data and graphs are provided to complement the two case studies. (PDF) [file pone.0284736.s002.pdf]

Across atoms to crossing continents: Application of similarity measures to biological location data - Supplementary Material

Fabian Schuhmann[1], Leonie Ryvkin[2,3,¤], James D. McLaren[4], Luca Gerhards[1], Ilia A. Solov'yov*[1,5,6],

1 Department of Physics, Carl von Ossietzky Universität Oldenburg, Carl-von-Ossietzky Str. 9-11, 26129 Oldenburg, Germany
2 Department of Mathematics & Computer Science, Technische Universiteit Eindhoven, 5612 AZ Eindhoven, Netherlands
3 Department of Computer Science, Ruhr-Universität Bochum, Universitätsstr. 150, 44801 Bochum, Germany
4 Institute of Chemistry and Marine Biology, Carl von Ossietzky Universität Oldenburg, Carl-von-Ossietzky Str. 9-11, 26129 Oldenburg, Germany
5 Research Centre for Neurosensory Science, Carl von Ossietzky Universität Oldenburg, Carl-von-Ossietzky Str. 9-11, 26129 Oldenburg, Germany
6 Center for Nanoscale Dynamics (CENAD), Carl von Ossietzky Universität Oldenburg, Ammerländer Heerstr. 114-118, 26129 Oldenburg, Germany

¤Current Address: Department of Mathematics & Computer Science, Technische Universiteit Eindhoven, 5612 AZ Eindhoven, Netherlands
* ilia.solovyov@uni-oldenburg.de

S2 Additional Case Study Information

Simulation methods for the protein case study

All simulations were set up using the online platform VIKING [15]. Parameters were kept identical to the earlier study [9], in which the CHARMM36 force field with CMAP corrections was used to describe the interatomic interactions [16–23]. The parameters for FAD and $FAD^{\bullet-}$ were adopted from a study by Xu *et al* [24]. The simulations were conducted using NAMD [25, 26] in an NPT (constant number of particles, pressure, and temperature) statistical ensemble at a temperature of 310 K and an atmospheric pressure of 1 bar.

All simulations were conducted for 200 ns. Establishing a base for comparison the original inactive DS and the RPD state were prolongated without any change to their respective redox charges. Two replica simulations were initiated starting from the original RPD conformation, in which the charges were reverted to the inactive DS form. The simulations are named Reverted1 and Reverted2.

The molecular dynamics simulation trajectories delivered the positions of all atoms in the ClCry4 protein for each snapshot in time. In order to remove global rotation and translation motions, the protein structures were aligned in an iterative process to minimize the RMSD: The whole structure of the protein was aligned to its first frame using Kabsch-Algorithm [27]. Subsequently, the structures are aligned again to the average structure after the first alignment. After each step, the RMSD was measured. The structure was considered aligned once the RMSD value reached a threshold.

Additional data for protein case study

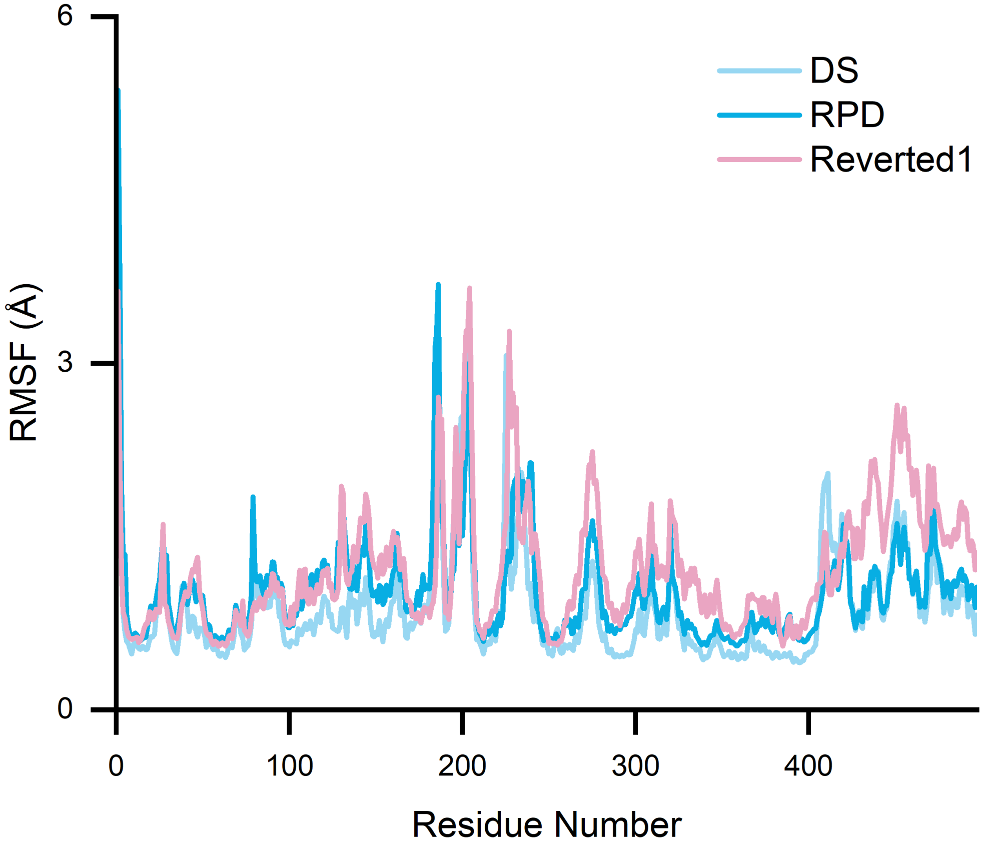

Fig S1. Root mean square fluctuation (RMSF) for Pigeon Crytochrome 4. The RMSF quantifies the internal versatility of a protein structure during a simulation trajectory without comparing to another trajectory. Its value for residue i is given by $RMSF(i) = \sqrt{\frac{1}{T}\sum_{t=1}^{T}\|r_{i,t} - r_{i,ref}\|^2}$, where T is the number of simulation snapshots and $r_{i,t}$ is the position of the Cα atom of residue i at snapshot t, the reference position is defined as $r_{i,ref} = \frac{1}{T}\sum_{t=1}^{T} r_{i,t}$. The higher the value, the greater the residues' location deviates from the configuration of the first snapshot. The DS is colored light blue, the RPD state in dark blue. The Reverted1 trajectory is colored to stand out in red. The region around residues 180-205 and residues 220-245 stand out, as well as the C-terminal region (residues 400+).

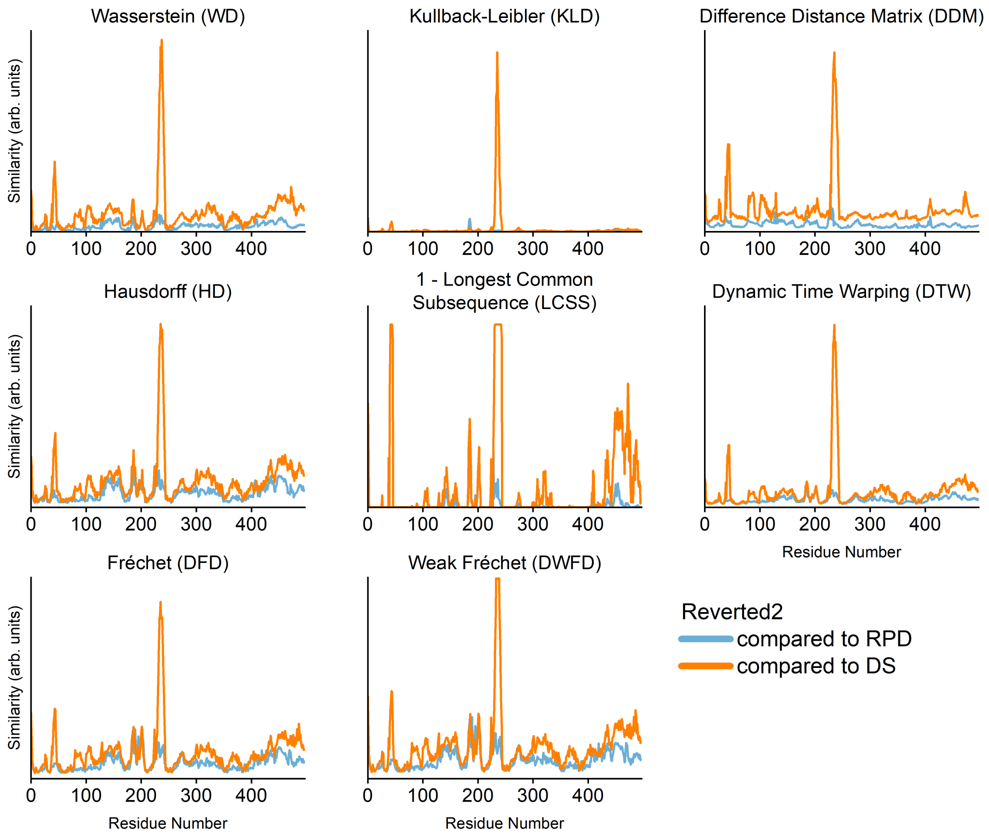

Fig S2. Similarity Measures for Pigeon Cryptochrome 4 - Replica Simulation. As already visualized in Fig. 2, the replica simulation shows the peak for each discussed similarity measure at residues 220 to 245. The ups and downs for each measure behave analogously as for the simulation trajectories discussed in the main paper.

Additional bird trajectories

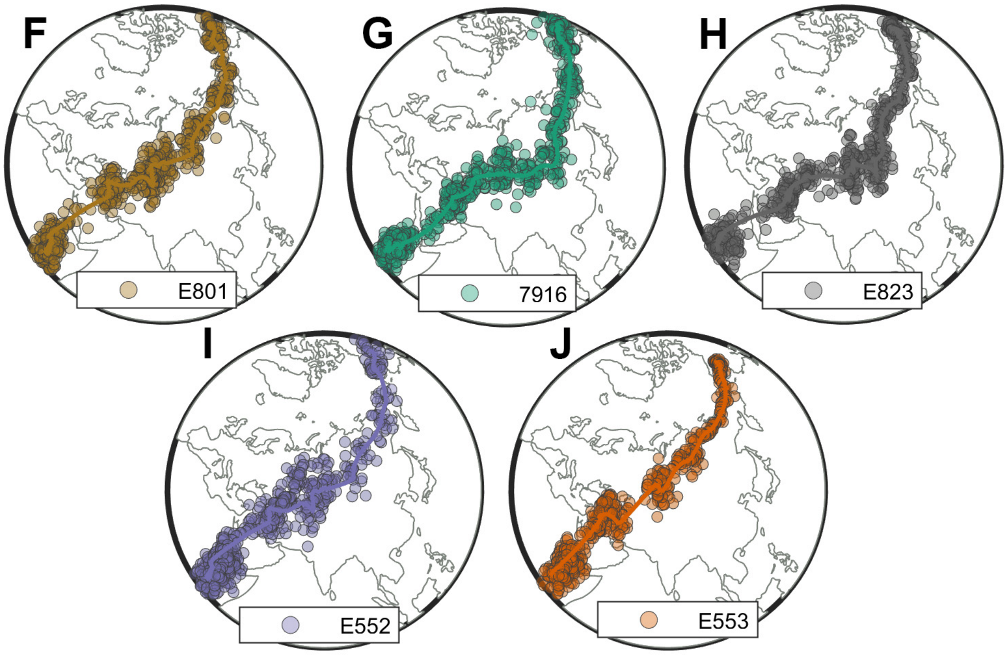

Fig S3. The remaining 5 bird flight trajectories supplementing Fig. 4 in the main paper. Panels A to E show the remaining 5 out of 8 bird trajectories. The maps are made with Natural Earth.

References

1. Cormen TH, Leiserson CE, Rivest RL, Stein C. Introduction to Algorithms, Third Edition. Cambridge, MA: The MIT Press; 2009.

2. Alt H, Godau M. Computing the Fréchet distance between two polygonal curves. Internat J Comput Geom Appl. 1995;5(1-2):75–91.

3. Tao Y, Both A, Silveira RI, Buchin K, Sijben S, Purves R, et al. A comparative analysis of trajectory similarity measures. GIScience & Remote Sensing. 2021;58:643 – 669.

4. Buchin M, Kilgus B, Kölzsch, A. Group diagrams for representing trajectories. International Journal of Geographical Information Science. 2020;34(12):2401–2433. doi:10.1080/13658816.2019.1684498.

5. Eiter T, Mannila H. Computing discrete Fr{é}chet distance. 1994 [cited 2022 November 21]. Available from: http://www.kr.tuwien.ac.at/staff/eiter/et-archive/cdtr9464.pdf

6. Sakoe H, Chiba S. Dynamic programming algorithm optimization for spoken word recognition. IEEE Transactions on Acoustics, Speech, and Signal Processing. 1978;26(1):43–49. doi:10.1109/TASSP.1978.1163055.

7. Chen L, Özsu M, Oria V. Robust and fast similarity search for moving object trajectories. Proceedings of the ACM SIGMOD International Conference on Management of Data. 2005; 491–502. doi:10.1145/1066157.1066213.

8. Rotter P, Skulimowski A, Kotropoulos C, Pitas I. Fast shape matching with the Hausdorff distance for pixel-represented objects. Mirage 2005: computer vision/computer graphics collaboration, techniques and applications: March, 1–2, 2005, INRIA Rocquencourt, France. 2005; pp. 205–211.

9. Schuhmann F, Kattnig DR, Solov'yov IA. Exploring Post-activation Conformational Changes in Pigeon Cryptochrome 4. J Phys Chem B. 2021;125(34):9652–9659. doi:10.1021/acs.jpcb.1c02795.

10. Kattnig DR, Nielsen C, Solov'yov IA. Molecular Dynamics Simulations Disclose Early Stages of the Photo-Activation of Cryptochrome 4. New J Phys. 2018;20(8):083018. doi:10.1088/1367-2630/aad70f.

11. Alt H, Braß P, Godau M, Knauer C, Wenk C. Computing the Hausdorff Distance of Geometric Patterns and Shapes. In: Aronov B, Basu S, Pach J, Sharir M, editors. Berlin, Heidelberg: Springer Berlin Heidelberg; 2003. p. 65–76. Available from: https://doi.org/10.1007/978-3-642-55566-4_4.

12. Orlova DY MSMCWJGEFAKGGYTSMWMRHLWG Zimmerman N. Earth Mover's Distance (EMD): A True Metric for Comparing Biomarker Expression Levels in Cell Populations. PLoS One. 2016;11(3):e0151859. doi:10.1371/journal.pone.0151859.

13. Edmonds J, Karp RM. Theoretical Improvements in Algorithmic Efficiency for Network Flow Problems. J ACM. 1972;19(2):248–264. doi:10.1145/321694.321699.

14. Kullback S, Leibler RA. On Information and Sufficiency. The Annals of Mathematical Statistics. 1951;22(1):79 – 86. doi:10.1214/aoms/1177729694.

15. Korol V, Husen P, Sjulstok E, Nielsen C, Friis I, Frederiksen A, et al. Introducing VIKING: A Novel Online Platform for Multiscale Modeling. ACS Omega. 2020;5(2):1254–1260. doi:10.1021/acsomega.9b03802.

16. Foloppe N, MacKerell AD. All-Atom Empirical Force Field for Nucleic Acids: I. Parameter Optimization Based on Small Molecule and Condensed Phase Macromolecular Target Data. J Comput Chem. 2000;21(2):86–104. doi:10.1002/(SICI)1096-987X(20000130)21:2¡86::AID-JCC2¿3.0.CO;2-G.

17. Best RB, Zhu X, Shim J, Lopes PEM, Mittal J, Feig M, et al. Optimization of the Additive CHARMM All-Atom Protein Force Field Targeting Improved Sampling of the Backbone ϕ, ψ and Side-Chain $\chi1$ and $\chi2$ Dihedral Angles. J Chem Theory Comput. 2012;8(9):3257–3273. doi:10.1021/ct300400x.

18. Hart K, Foloppe N, Baker CM, Denning EJ, Nilsson L, MacKerell AD. Optimization of the CHARMM Additive Force Field for DNA: Improved Treatment of the BI/BII Conformational Equilibrium. J Chem Theory Comput. 2012;8(1):348–362. doi:10.1021/ct200723y.

19. Pavelites JJ, Gao J, Bash PA. A Molecular Mechanics Force Field for NAD+ , NADH, and the Pyrophosphate Groups of Nucleotides. J Comput Chem. 1996;18(2):221–239.

20. MacKerell AD, Banavali NK. All-Atom Empirical Force Field for Nucleic Acids: II. Application to Molecular Dynamics Simulations of DNA and RNA in Solution. J Comput Chem. 2000;21(2):105–120. doi:10.1002/(SICI)1096-987X(20000130)21:2¡105::AID-JCC3¿3.0.CO;2-P.

21. Denning EJ, Priyakumar UD, Nilsson L, MacKerell AD. Impact of 20-Hydroxyl Sampling on the Conformational Properties of RNA: Update of the CHARMM All-Atom Additive Force Field for RNA. J Comput Chem. 2011;32(9):1929–1943. doi:10.1002/jcc.21777.

22. MacKerell AD, Feig M, Brooks CL. Improved Treatment of the Protein Backbone in Empirical Force Fields. J Am Chem Soc. 2004;126(3):698–699. doi:10.1021/ja036959e.

23. MacKerell AD, Bashford D, Bellott M, Dunbrack RL, Evanseck JD, Field MJ, et al. All-atom Empirical Potential for Molecular Modeling and Dynamics Studies of Proteins. J Phys Chem B. 1998;102(18):3586–3616. doi:10.1021/jp973084f.

24. Xu J, Jarocha LE, Zollitsch T, Konowalczyk M, Henbest KB, Richert S, et al. Magnetic sensitivity of cryptochrome 4 from a migratory songbird. Nature. 2021;594(7864):535–540. doi:10.1038/s41586-021-03618-9.

25. Phillips JC, Braun R, Wang W, Gumbart J, Tajkhorshid E, Villa E, et al. Scalable Molecular Dynamics with NAMD. J Comput Chem. 2005;26(16):1781–1802. doi:10.1002/jcc.20289.

26. Phillips JC, Hardy DJ, Maia JDC, Stone JE, Ribeiro JV, Bernardi RC, et al. Scalable Molecular Dynamics on CPU and GPU Architectures with NAMD. J Chem Phys. 2020;153(4):44130. doi:10.1063/5.0014475.

27. Kabsch W. A discussion of the solution for the best rotation to relate two sets of vectors. Acta Crystallogr Sect A. 1978;34(5):827–828. doi:10.1107/S0567739478001680.
