# Supplementary material for: Across atoms to crossing continents: Application of similarity measures to biological location data
Source: PLoS One. 2023 May 15;18(5):e0284736. doi: 10.1371/journal.pone.0284736 (PMC10184918; doi:10.1371/journal.pone.0284736)
Supplement: S3 File — (PDF) [file pone.0284736.s003.pdf]

# Across atoms to crossing continents: Application of similarity measures to biological location data - Supplementary Material

Fabian Schuhmann[1], Leonie Ryvkin[2,3,¤], James D. McLaren[4], Luca Gerhards[1], Ilia A. Solov'yov*[1,5,6],

**1** Department of Physics, Carl von Ossietzky Universität Oldenburg, Carl-von-Ossietzky Str. 9-11, 26129 Oldenburg, Germany
**2** Department of Mathematics & Computer Science, Technische Universiteit Eindhoven, 5612 AZ Eindhoven, Netherlands
**3** Department of Computer Science, Ruhr-Universität Bochum, Universitätsstr. 150, 44801 Bochum, Germany
**4** Institute of Chemistry and Marine Biology, Carl von Ossietzky Universität Oldenburg, Carl-von-Ossietzky Str. 9-11, 26129 Oldenburg, Germany
**5** Research Centre for Neurosensory Science, Carl von Ossietzky Universität Oldenburg, Carl-von-Ossietzky Str. 9-11, 26129 Oldenburg, Germany
**6** Center for Nanoscale Dynamics (CENAD), Carl von Ossietzky Universität Oldenburg, Ammerländer Heerstr. 114-118, 26129 Oldenburg, Germany

¤Current Address: Department of Mathematics & Computer Science, Technische Universiteit Eindhoven, 5612 AZ Eindhoven, Netherlands
* ilia.solovyov@uni-oldenburg.de

## S3 Code Example for SiMBols

In order to supply some guideline on how the python package SiMBols and the preprocesses for protein data can be utilized, a commented ready-to-run example workflow is provided. Needed input data, as well as this example, can be downloaded and tested at *https://gitlab.uni-oldenburg.de/quantbiolab/simbols*.

```
import SiMBols

"""We show a short example through all the steps for two protein
    trajectories. For this case, two example trajectories of Pigeon
    Cryptochrome 4 in a so-called Darkstate and a Radical Pair D state
    were truncated to just 200 snapshots. This allows for a fast exemplary
     computation. The necessary namd simulation files (.psf and .dcd)
    files are also provided to allow a trial run of the methods provided
    in the package. We also measure the time, the example output shows the
     time it took on Intel(R) Core(TM) i5-8250U CPU @ 1.60GHz. It will be
    running on one CPU only, unless specified"""

print("Begin Example Output:")
import time

start = time.perf_counter()  # Set a start time to measure how long it
    takes

"""Preparation: Preparation reads a .psf and a .dcd file to transfer the
    location data of all the atoms to a numpy array. It takes a file
    location as string for the .psf, and a list of file locations as
    string for the dcd. This allows to load multiple .dcds. Note however,
    also a single .dcd needs to be supplied in a list with one element."""
```

```
"""We give the necessary paths:"""
traj1_psf = 'trajectory1.psf'  # This is the dark state trajectory psf.
traj1_dcd = ['trajectory1.dcd']  # This is the dark state trajectory dcd.
traj2_psf = 'trajectory2.psf'  # This is the radical pair D state
    trajectory psf.
traj2_dcd = ['trajectory2.dcd']  # This is the radical pair D state
    trajectory dcd.

"""Preparation.getList() returns a numpy array of shape (timesteps, atoms,
     3) containing the location of each atom in 3d space. It will, however
    , remove water and ions. The second array is of shape (atoms) and will
     contain the names for each atom as an mdtraj atom object. To actually
     read the names, it is good to cast to string. The last return is a
    frame object from mdtraj. It is returned just in case to allow vmd
    like selection language later on. As we do not plan to skip any frames
     or use a stride to skip frames, we do not need to provide these
    optional arguments. At this stage, we pretend to not know how many
    frames we actually have available, so, even though it is costly, we
    want getList() to count the number of frames for us. Lastly, we supply
     a name, as getList() will do a backup save as a .npy numpy array."""

import ProteinPreprocessing.Preparation as Preparation

traj_1, names_1, chunk_1 = Preparation.getList(dcds=traj1_dcd, psf=
    traj1_psf, name="trajectory1")
traj_2, names_2, chunk_2 = Preparation.getList(dcds=traj2_dcd, psf=
    traj2_psf, name="trajectory2")

preparation = time.perf_counter()  # Time after preparation is done

"""Alignment: Protein structures are subject to internal motions during a
    simulation and can also rotate or even move as a whole. These motions
    will distort the later similarity measures. In order to only look at
    the internal motions and rearrangements, the protein structures need
    to be aligned individually and relative to each other. This alignment
    is done by Alignment.Align_states(), which returns two numpy arrays of
     the same form as Preparation.getList(). At the very least,
    Align_states takes both trajectory arrays and both name arrays, as
    returned by getList(). We set an accuracy of 0.03, so residues are
    considered, if their RMSF is lower than 3nm. We, once more, only need
    the standard input and can take the default for the rest. We have a
    small data set, so we can set Workers to the default of 1 for the RMSF
     calculation. No need to parallalize here. Lastly, we do not want to
    align according to a specific selection or want to have a specifially
    good alignment, so we can also keep the RMSD threshold as default."""


import ProteinPreprocessing.Alignment as Alignment

traj1_aligned, traj2_aligned = Alignment.Align_states(traj_1, traj_2,
    names_1, names_2, accuracy=0.05)

alignment = time.perf_counter()  # Time after alignment is done

"""Now, we make sure, that the trajectories have the right form and right
    length for the actual measures. The measures themselves, as they are
    made for each residue take numpy arrays of shape (atoms, timestep, 3)
    as opposed to the getList() or Alignment output of shape (timestep,
    atoms, 3). Additionally, we get another chance to input a Stride value
     to limit our dataset for test purposes, for instance. We can also set
     a number of frames, if we want to make sure, that both trajectories
    have the same length. As we continue with the changed data only, we
    can overwrite our old aligned numpy array with the transposed ones. We
     start out by preparing the selection on which we really want to
    compute our measures. Here, we chose to look at the CA atom in each
```

```
    amino acids backbone."""

traj1_index = Alignment.get_limited_selection_list(names_1, ["CA"])
traj2_index = Alignment.get_limited_selection_list(names_2, ["CA"])

"""Now we got all the inputs for SiMBols and we can create two Trajectory
    Objects. These Objects are still in the wrong form, so they need to be
     adjusted using the otc method (objects, time, coordinates). At the
    moment the array is still sorted as (time, objects, coordinates). We
    are also providing an index as calculated above, so SiMBols knows,
    whether to consider all objects."""

traj1_aligned = SiMBols.Trajectory(array=traj1_aligned, index=traj1_index)
traj2_aligned = SiMBols.Trajectory(array=traj2_aligned, index=traj2_index)



traj1_aligned.otc(transpose=True)
traj2_aligned.otc(transpose=True)


"""Now we are ready to calculate the first distance measures. We supply
    the two trajectories to the measures and additionally supply a number
    of Workers for parallalized tasks to allow a quicker calculation. In
    this example, we will calculate the Wasserstein distance (wd) and the
    Frechet distnace (dfd).
We start by creating a comparer and give the two trajectories to it. The
    Comparer class has some built in functions to check the sanity of the
    input. We will forgo this check in the example"""

comp = SiMBols.Comparer(traj1_aligned, traj2_aligned)
comp.make_Check()
comp.cut()

print("And so it begins...")

comp.dfd(Workers=4)  # Calcuate the frechet distance on 4 processes
comp.wd()  # Calculate the Wasserstein distance

comp.save_all() # Save the results as numpy arrays

distances = time.perf_counter()  # Time after measures are done is done

print(f"Preparation: {round(preparation - start, 2)} second(s)!")
print(f"Alignment: {round(alignment - preparation, 2)} second(s)!")
print(f"Distances: {round(distances - alignment, 2)} second(s)!")
print(f"Total: {round(distances - start, 2)} second(s)!")

print("End Example Output")
```
